# Antagonistic Functions of USAG-1 and RUNX2 during Tooth Development

**DOI:** 10.1371/journal.pone.0161067

**Published:** 2016-08-12

**Authors:** Yumiko Togo, Katsu Takahashi, Kazuyuki Saito, Honoka Kiso, Hiroko Tsukamoto, Boyen Huang, Motoko Yanagita, Manabu Sugai, Hidemitsu Harada, Toshihisa Komori, Akira Shimizu, Mary MacDougall, Kazuhisa Bessho

**Affiliations:** 1 Department of Oral and Maxillofacial Surgery, Graduate School of Medicine, Kyoto University, 54 Shogoin-Kawahara-cho, Sakyo-ku, 606–8507, Kyoto, Japan; 2 School of Dentistry and Health Sciences, Faculty of Science, Charles Sturt University, Orange, NSW, 2800, Australia; 3 Department of Nephrology, Graduate School of Medicine, Kyoto University, 54 Shogoin-Kawahara-cho, Sakyo-ku, 606–8507, Kyoto, Japan; 4 Department of Molecular Genetics, Division of Medicine, Faculty of Medical Sciences, University of Fukui, Matsuokashimoaizuki, Eiheiji-cho, Yoshida-gun, Fukui, 910–1193, Japan; 5 Division of Developmental Biology and Regenerative Medicine, Department of Anatomy, Iwate Medical University, 2-1-1, Nishitokuta, Yahaba, Iwate, 028–3694, Japan; 6 Department of Cell Biology, Unit of Basic Medical Sciences, Nagasaki University Graduate School of Biomedical Sciences, 1-7-1 Sakamoto, Nagasaki, 852–8588, Japan; 7 Department of Experimental Therapeutics, Institute for Advancement of Clinical and Translational Science, Kyoto University Hospital, 54 Shogoin-Kawahara-cho, Sakyo-ku, 606–8507, Kyoto, Japan; 8 Institute of Oral Health Research, Department of Oral and Maxillofacial Surgery, School of Dentistry, University of Alabama, Birmingham, Alabama, United States of America; Universiteit Gent, BELGIUM

## Abstract

Supernumerary teeth and tooth agenesis are common morphological anomalies in humans. We previously obtained evidence that supernumerary maxillary incisors form as a result of the successive development of the rudimentary maxillary incisor tooth germ in *Usag-1* null mice. The development of tooth germs is arrested in *Runx2* null mice, and such mice also exhibit lingual epithelial buds associated with the upper molars and incisors. The aim of this study is to investigate the potential crosstalk between *Usag-1* and *Runx2* during tooth development. In the present study, three interesting phenomena were observed in double null *Usag-1*^-/-^/*Runx2*^-/-^ mice: the prevalence of supernumerary teeth was lower than in *Usag-1* null mice; tooth development progressed further compared than in *Runx2* null mice; and the frequency of molar lingual buds was lower than in *Runx2* null mice. Therefore, we suggest that RUNX2 and USAG-1 act in an antagonistic manner. The lingual bud was completely filled with odontogenic epithelial Sox2-positive cells in the *Usag-1*^+/+^/*Runx2*^-/-^ mice, whereas almost no odontogenic epithelial Sox2-positive cells contributed to supernumerary tooth formation in the rudimentary maxillary incisors of the *Usag-1*^-/-^/*Runx2*^+/+^ mice. Our findings suggest that RUNX2 directly or indirectly prevents the differentiation and/or proliferation of odontogenic epithelial Sox2-positive cells. We hypothesize that RUNX2 inhibits the bone morphogenetic protein (BMP) and/or Wnt signaling pathways regulated by USAG-1, whereas RUNX2 expression is induced by BMP signaling independently of USAG-1.

## Introduction

Alterations in tooth development can lead to numerous dental anomalies, with supernumerary teeth (extra teeth) and tooth agenesis (missing teeth) being among the most common morphological anomalies seen in humans. A number of mouse mutant models have provided insights into the formation of supernumerary teeth [1–14]. Mice, unlike humans, have continuously erupting incisors and three molars that are separated by a tooth formation-free region called the diastema. Several mechanisms have been proposed to account for the formation of supernumerary teeth in mice [15, 16]. One plausible explanation for supernumerary tooth formation is the rescue of tooth rudiments in the diastema or maxillary deciduous incisors [9, 17, 18]. USAG-1 (also known as Sostdc1, ectodin, and Wise) is a bone morphogenetic protein (BMP) antagonist [19, 20, 21]. We have demonstrated that the inhibition of apoptosis can lead to the successive development of the rudimentary maxillary incisors in *Usag-1* null mice [9, 15, 22]. Furthermore, in a *Usag-1*-deficient mouse model increased BMP signaling was found to prevent apoptosis, leading to the development of supernumerary teeth [15]. In particular, our previous study suggested that specific interactions between BMP-7 and USAG-1 regulate supernumerary rudimentary maxillary incisor formation [22]. There is plenty of evidence supporting a genetic etiology for tooth agenesis in the literature [23]. Various molecules that are essential for the early stages of tooth formation (in the dental lamina, bud, cap), such as Pax9, Msx1, Lef1, and Runx2, have been identified through analyses of specific knockout mice [24, 25, 26, 27]. Tooth development arrests at the bud stage in *Runx2*-deficient mice [27], while heterozygous mutations in *RUNX2* cause the human disorder cleidocranial dysplasia (CCD), which is characterized by multiple supernumerary teeth [28]. Interestingly, *Runx2* null mice possess a lingual epithelial bud, which represents a putative successional tooth associated with the upper molars and incisors [29, 30].

In the maxillary incisor region, we have shown using *in situ* hybridization and lacZ staining that *Usag-1* mRNA is expressed in the rudiment and normal maxillary incisors [19]. *Usag-1* expression is also found in the forming molar region that functions to regulate tooth cusp patterning [31]. RUNX2 is a transcription factor that is essential for both bone and tooth formation and has three isoforms (types I-III). During tooth development, all three RUNX2 isoforms are expressed in the dental epithelium and/or mesenchyme of both the incisors and molars and exhibit distinct temporospatial patterns [27, 32–34].

Previous studies have demonstrated that *Sox2* mRNA is expressed in dental epithelial cells, particularly in the labial cervical loop area of the mouse incisor, which contains dental epithelial stem cells. Sox2 has been shown to play important roles in maintaining the multipotency of these and other stem cells [35–38]. However, it remains unclear whether *Sox2* expression is involved in the progressive development of rudimentary maxillary incisors, lingual bud formation, or the formation of supernumerary teeth in *Usag-1* and *Runx2* null mice.

The purpose of this study is to investigate the potential crosstalk between *Usag-1* and *Runx2* to determine whether they act in an antagonistic or synergic manner during tooth formation, developmental arrest, and supernumerary tooth formation using *Usag-1*^-/-^ and *Runx2*^-/-^ mice. Furthermore, we examine the contribution of dental epithelial Sox2-positive cells to supernumerary maxillary incisor formation and lingual bud formation in these mouse models.

## Materials and Methods

### Ethics statement

All procedures were approved by the animal care committee at Kyoto University, Japan.

### Mouse strains

*Usag-1* null mice (a supernumerary teeth mouse model) [20] and *Runx2* null mice (a tooth agenesis model) [39] with a C57Bl6/J background were used in this study. Double *Usag-1*^-/-^ and *Runx2*^-/-^ knockout mice were generated by crossing two independent mouse null lines with the control wild-type mice. Timed-pregnant embryos, in which day 0 (E0) was considered to start at midnight on the day prior to the detection of a vaginal plug, were used for the experiments. Their mice were sacrificed with CO^2^ gas.

### Analysis of tooth phenotypes

At various timepoints from embryonic day 15 (E15) to postnatal day 0 (P0), the heads of pups of each genotype were fixed in 10% formalin and embedded in paraffin. Serial sections (7 μm) of the pups' heads were obtained. The sections were stained with hematoxylin and eosin, and the morphology of the maxillary and mandibular incisors and molars was documented.

### Immunohistochemistry

The E12-E15 mouse heads were also subjected to standard immunohistochemical examinations. The tissues were fixed in 4% paraformaldehyde, embedded in paraffin, and serially sectioned (7 μm). The sections were incubated with primary rabbit monoclonal antibodies against Sox2 (Ab92494) at a 1:100 dilution (Abcam, Japan) followed by secondary goat and mouse anti-rabbit antibodies (Nichirei Bioscience, Japan). The sections were then counterstained with hematoxylin and dehydrated in a graded series of ethanol and xylene. Finally, coverslips were applied, and the sections were viewed under a microscope.

## Results

### Abrogation of *Usag-1* expression rescues hypoplastic and poorly differentiated molar and incisor phenotypes in *Runx2*^-/-^ mice

**Fig 1:**
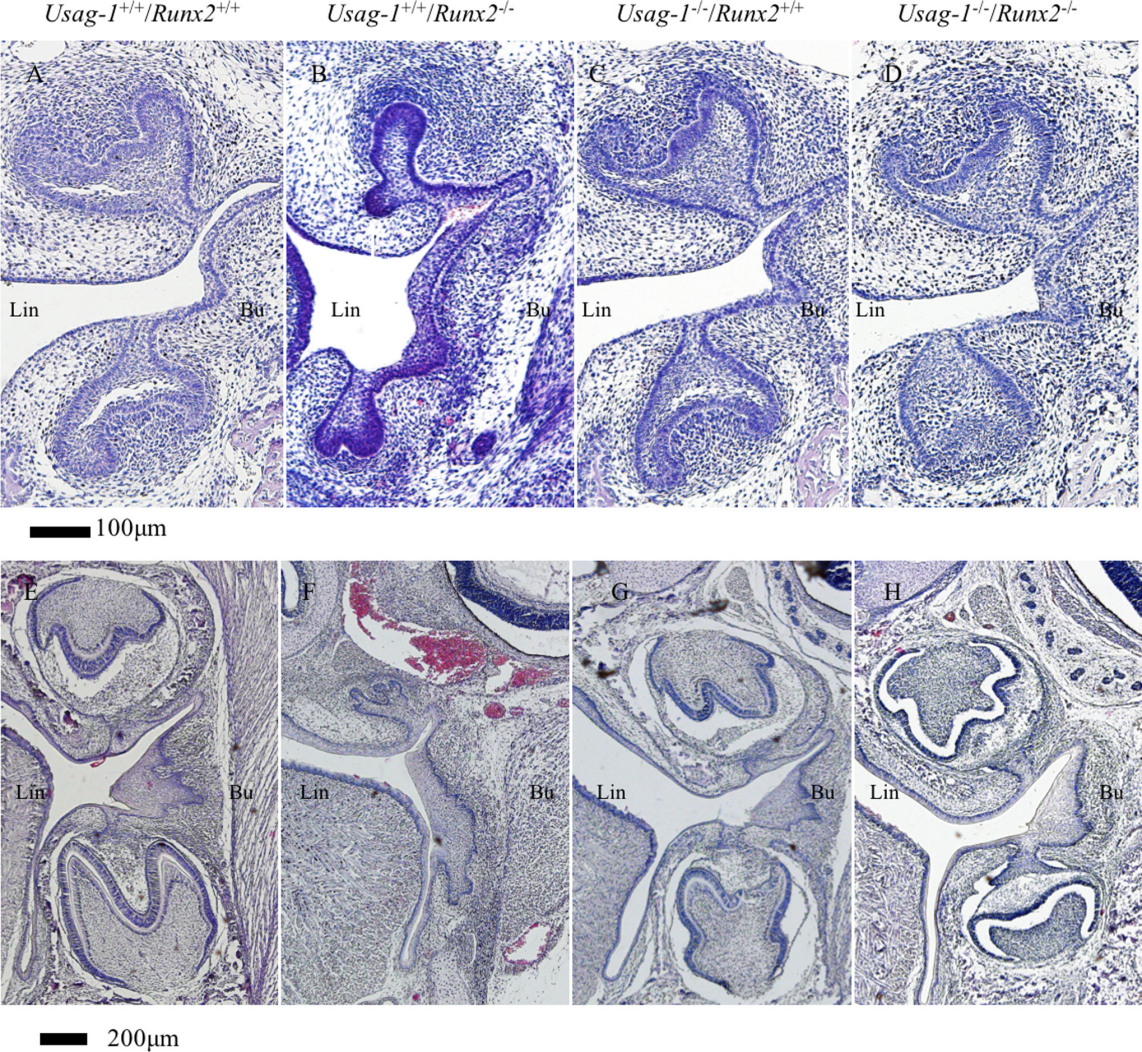
**Histological analysis (H&E staining) of frontal sections of molar teeth from E15 (A-D) and P0 (E-H) mice.** At E15, the molars of the *Usag-1*^+/+^/*Runx2*^-/-^ mice (B) were at the bud stage while the molars of all the other mice were at the late cap stage. The *Usag-1*^+/+^/*Runx2*^-/-^ mouse molars each exhibited a distinct lingual bud (B, arrow). At P0, morphological examinations indicated that molar tooth development had arrested in the *Usag-1*^+/+^/*Runx2*^-/-^ mice (F) compared with the other transgenic mouse lines (G & H), which displayed similar molar tooth morphology to the wild-type mice (E). Lin: Lingual side.

**Fig 2:**
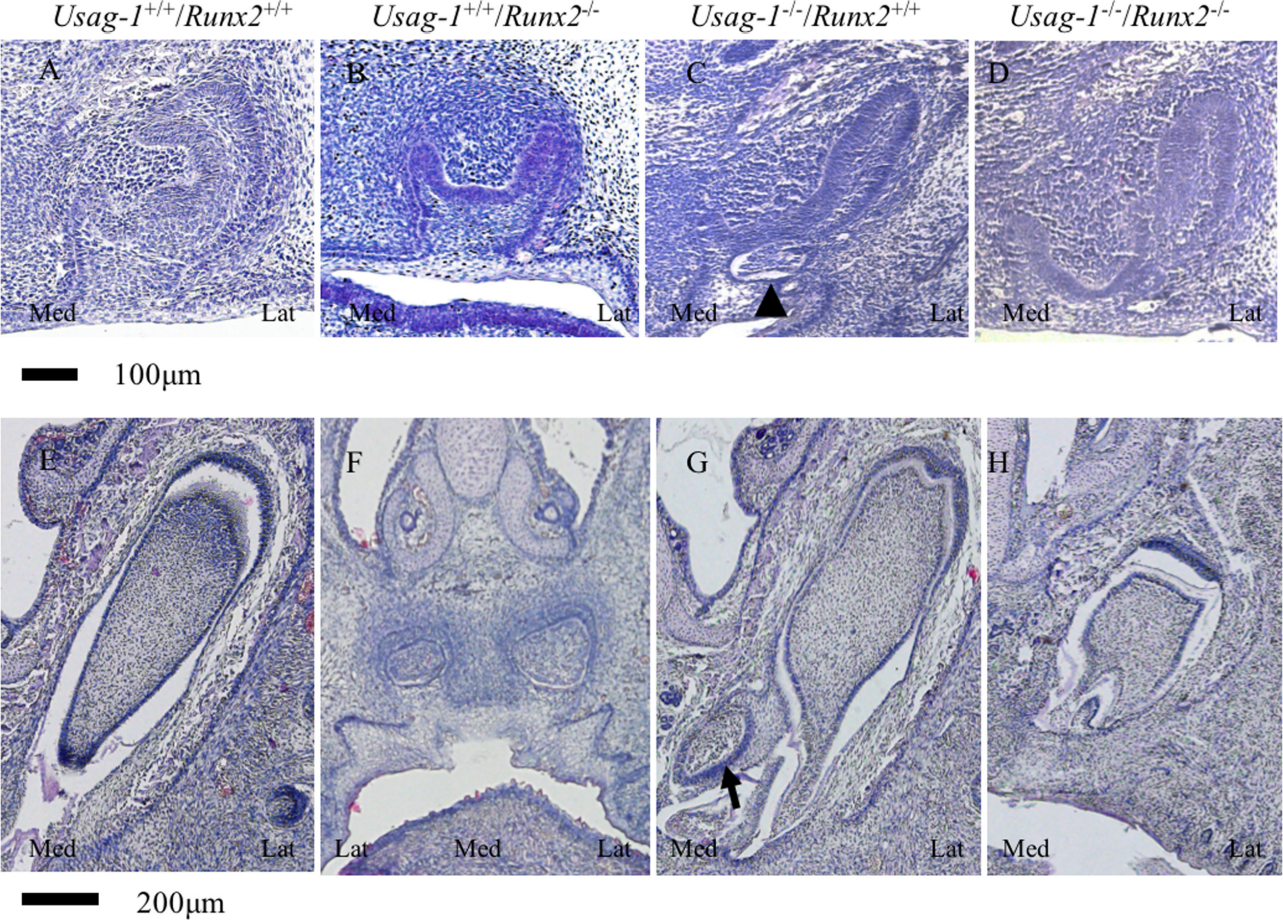
**Histological analysis (H&E staining) of frontal sections of maxillary incisors from E15 (A-D) and P0 (E-H) mice.** At E15, the incisors of the *Usag-1*^+/+^/*Runx2*^-/-^ mice were at the cap stage whereas those of the other transgenic mouse lines were at the late cap stage. The arrowhead indicates a rudimentary tooth germ in the maxillary incisor of a *Usag-1*^-/-^/*Runx2*^+/+^mouse (C). At P0, the incisors of the *Usag-1*^+/+^/*Runx2*^-/-^ mice (F) were in a state of developmental arrest compared with those of the normal wild-type mice (E). The arrow indicates a supernumerary tooth (G) in a *Usag-1*^-/-^/*Runx2*^+/+^mouse.

**Fig 3:**
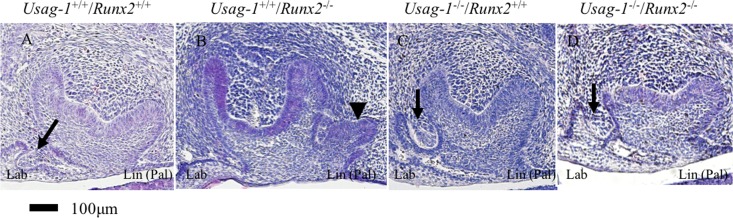
**Histological analysis (H&E staining) of sagittal sections of maxillary incisors at E15 (A-D).** The arrows indicate rudimentary tooth germs in the maxillary incisors of *Usag-1*^+/+^/*Runx2*^+/+^(A), *Usag-1*^-/-^/*Runx2*^+/+^ (C), and *Usag-1*^-/-^/*Runx2*^-/-^ (D) mice. The arrowhead indicates a lingual bud that formed in the incisor of a *Usag-1*^+/+^/*Runx2*^-/-^ mouse (B).

**Fig 4:**
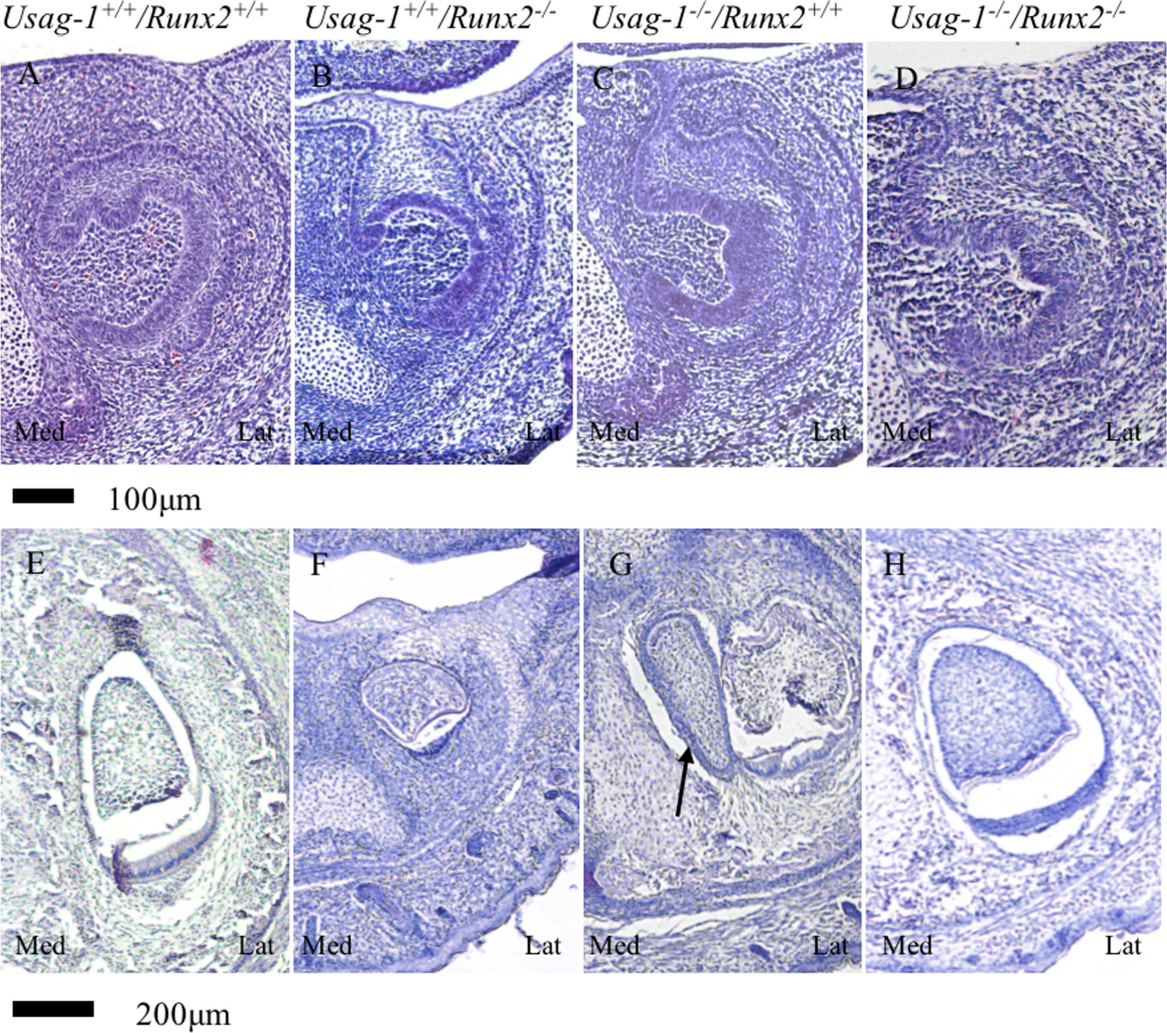
**Histological analysis (H&E staining) of frontal sections of mandibular incisors from E15 (A-D) and P0 (E-H) mice.** At E15, the incisors of the *Usag-1*^+/+^/*Runx2*^-/-^ mice were at the cap stage, whereas those of the other transgenic mouse lines were at the late cap stage. At P0, the incisor morphology of the *Usag-1*^+/+^/*Runx2*^-/-^ mice (F) was markedly different from that of the normal wild-type mice (E). The arrow indicates a supernumerary tooth (G) in a *Usag-1*^-/-^ mouse.

**Fig 5:**
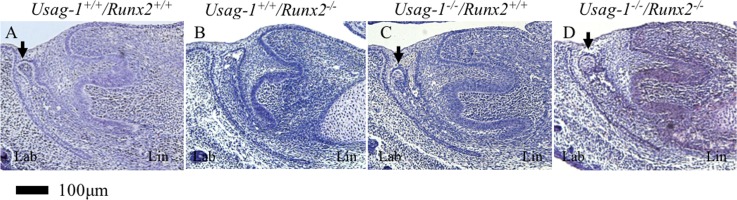
**Histological analysis (H&E staining) of sagittal sections of mandibular incisors at E15 (A-D).** The arrows indicate rudimentary tooth germs in the mandibular incisors of *Usag-1*^+/+^/*Runx2*^+/+^(A), *Usag-1*^-/-^/*Runx2*^+/+^ (C), and *Usag-1*^-/-^/*Runx2*^-/-^ (D) mice.

*Runx2* null mice die shortly after birth due to the absence of bone formation [39, 40]. To test the hypothesis that abrogating *Usag-1* expression has the potential to rescue arrested molar and incisor tooth formation in *Runx2*^-/-^ mice, we performed a series of histological investigations of *Usag-1*^+/+^/*Runx2*^+/+^, *Usag-1*^+/+^/*Runx2*^-/-^, *Usag-1*^-/-^/*Runx2*^+/+^, and *Usag-1*^-/-^/*Runx2*^-/-^ mice at E15 and P0. At E15, both the maxillary and mandibular molar tooth germs were at the bud stage of development in the *Usag-1*^+/+^/*Runx2*^-/-^ mice (Fig 1B). Interestingly, molar development progressed to the late cap stage (Fig 1D) or the cap stage in 50% of the examined *Usag-1*^-/-^/*Runx2*^-/-^ pups (Table 1). On the other hand, markedly arrested molar development was seen in the *Usag-1*^+/+^/*Runx2*^-/-^ mice, even at P0. In fact, their molars started to regress, and the tooth organ disappeared (Fig 1F). At P0, 25% of the *Usag-1*^-/-^/*Runx2*^*-/-*^ mice presented with molars that were at an appropriate stage of development and exhibited a normal morphology (Fig 1H, Table 1), and molar development progressed to the bell stage in 75% of the *Usag-1*^-/-^/*Runx2*^*-/-*^ mice (Table 1). Histological analyses of the maxillary and mandibular incisor tooth primordia obtained from the *Usag-1*^-/-^/*Runx2*^-/-^ mice at E15 and P0 demonstrated a rescued phenotype (Figs 2D and 2H, 3D, 4D and 4H and 5D), while the incisor tooth primordia from the *Usag-1*^+/+^/*Runx2*^-/-^ mice displayed less severe developmental delays than the molars of the same mice (Figs 2B and 2F, 3B, 4B and 4F and 5B). The rescue of the molar phenotype was much more complete than the rescue of the incisor phenotype, although molar rescue and incisor rescue occurred at similar frequencies (Table 1). These results demonstrate that abrogating *Usag-1* expression partially rescued the hypoplastic and poorly differentiated molar and incisor phenotypes of *Runx2*^-/-^ mice.

**Table 1:** The phenotypes of *Usag-1*^+/+^/*Runx2*^+/+^, *Usag-1*^+/+^/*Runx2*^-/-^, *Usag-1*^-/-^/*Runx2*^+/+^ and *Usag-1*^-/-^/*Runx2*^-/-^ mice at E15 and P0.

E15	*Usag-1*^*+/+*^*/Runx2*^*+/+*^	*Usag-1*^*+/+*^*/Runx2*^*-/-*^	*Usag-1*^*-/-*^*/Runx2*^*+/+*^	*Usag-1*^*-/-*^*/Runx2*^*-/-*^
number	5	6	6	6
Developmental stage of tooth germ (incisors)	Late cap stage	Cap stage	Late cap stage	Late cap stage 50%
				Cap stage 50%
Developmental stage of tooth germ (molars)	Late cap stage	Bud stage	Late cap stage	Late cap stage 50%
Formation of lingual buds	incisors 0%	incisors 100%	incisors 0%	incisors 50%
	molars 0%	molars 100%	molars 0%	molars 67%
Incidence rate of rudimentary incisor tooth germs	Maxilla 80%	Maxilla 0%	Maxilla 100%	Maxilla 83%
	Mandible 20%	Mandible 0%	Mandible 83%	Mandible 16%
P0	*Usag-1*^*+/+*^*/Runx2*^*+/+*^	*Usag-1*^*+/+*^*/Runx2*^*-/-*^	*Usag-1*^*-/-*^*/Runx2*^*+/+*^	*Usag-1*^*-/-*^*/Runx2*^*-/-*^
number	3	3	6	4
Tooth morphology (incisors)	Normal	Abnormal	Normal	Normal 25%
				Abnormal 75%
Tooth morphology (molars)	Normal	Developmental arrest	Normal	Normal 25%
				Bell stage 75%
Incidence rate of supernumerary teeth (incisor)	Maxilla 0%	Maxilla 0%	Maxilla 100%	Maxilla 25%
	Mandible 0%	Mandible 0%	Mandible 100%	Mandible 0%

### Abrogation of *Runx2* expression prevents supernumerary maxillary incisor formation in *Usag-1* null mice

Next, we investigated whether the abrogation of *Runx*2 expression would inhibit supernumerary maxillary rudimentary incisor formation in *Usag-1* null mice. We previously demonstrated that supernumerary maxillary incisors formed as a result of the successive development of the rudimentary incisor primordia [9]. At E15, the region from which the maxillary rudimentary incisors arose was identified at the labial border of the epithelial invagination in all of the mutant mice, except the *Usag-1*^+/+^/*Runx2*^-/-^ mice (Fig 3A–3D) (as described by [9, 41]). The frequency of rudimentary incisors was similar in all strains except *Usag-1*^+/+^/*Runx2*^-/-^ at E15 (Table 1). The rudimentary tooth primordia of the *Usag*-1^-/-^/*Runx2*^-/-^ mice regressed, and their size decreased from E15 to P0; however, these effects did not exhibit complete penetrance (Fig 2E–2H). The incidence of inhibited supernumerary formation was 75% (Table 1). The mandibular incisors of the *Usag*-1^-/-^/*Runx2*^-/-^ mice exhibited a similar phenotype (Figs 4 and 5 and Table 1). This means that abrogating *Runx2* expression partially inhibits supernumerary maxillary incisor formation in *Usag-1* null mice.

### Abrogation of *Usag-1* inhibits maxillary molar and incisor lingual bud formation in *Runx2*^-/-^ mice

At E15, the maxillary molar regions of the *Usag-1*^+/+^/*Runx2*^-/-^ mice contained a lingual bud (Fig 1B); i.e., an extra budding of the invaginating epithelium on the palatal side (lingual) of the normal bud. No lingual buds were seen in the *Usag-1*^+/+^/*Runx2*^+/+^ or *Usag-1*^-/-^/*Runx2*^+/+^ mice at E15 (Fig 1A and 1C). However, no lingual buds formed in 33% of the *Usag-1*^-/-^/*Runx2*^-/-^ mice (Fig 1D, Table 1). Lingual buds were also observed in the maxillary incisor tooth germs of the *Usag-1*^+/+^/*Runx2*^-/-^ mice at E15 (Fig 3B). In the *Usag-1*^-/-^/*Runx2*^-/-^ mice, maxillary incisal lingual bud formation occurred at a frequency of 50% (Table 1). These results demonstrated that abrogating *Usag-1* expression partially inhibited the lingual bud formation seen in the maxillary molars and incisors of *Runx2*^-/-^ mice.

### Odontogenic epithelial Sox2-positive cells contribute to lingual bud formation but not rudimental incisor development

**Fig 6:**
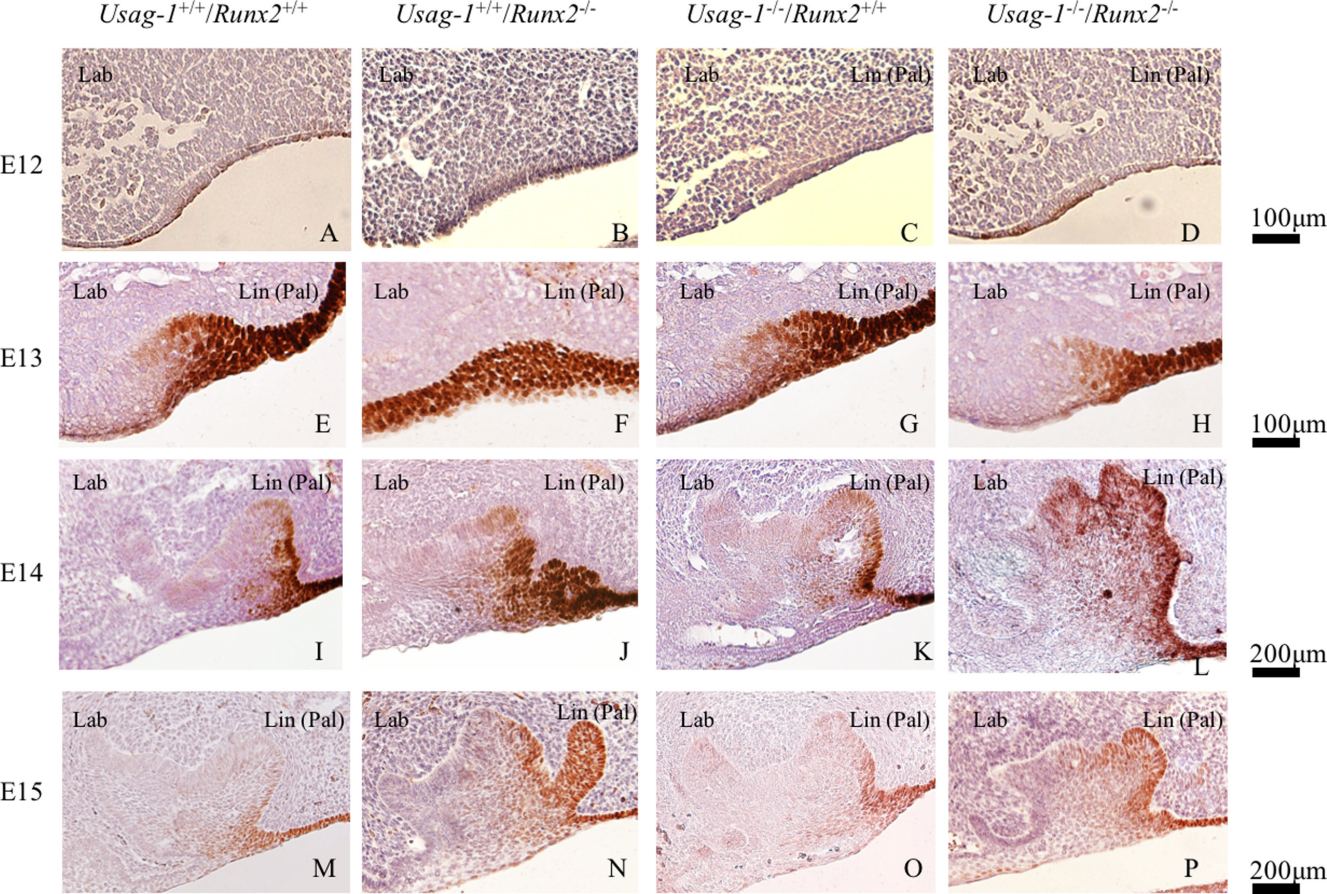
**SOX2 immunostaining in sagittal maxillary incisor sections from E12 (A-D), E13 (E-H), E14 (I-L), and E15 (M-P) embryos.** At E13, strong SOX2 staining was seen in the lingual region of the epithelial dental lamina in all mice (E, G & H) except for the *Usag-1*^+/+^/R*unx2*^-/-^ mice, in which SOX2 was found throughout the dental lamina (F). At E15, strong SOX2 staining was seen in the additional lingual bud in the *Usag-1*^*+/+*^/*Runx2*^*-/-*^ mice (N).

**Fig 7:**
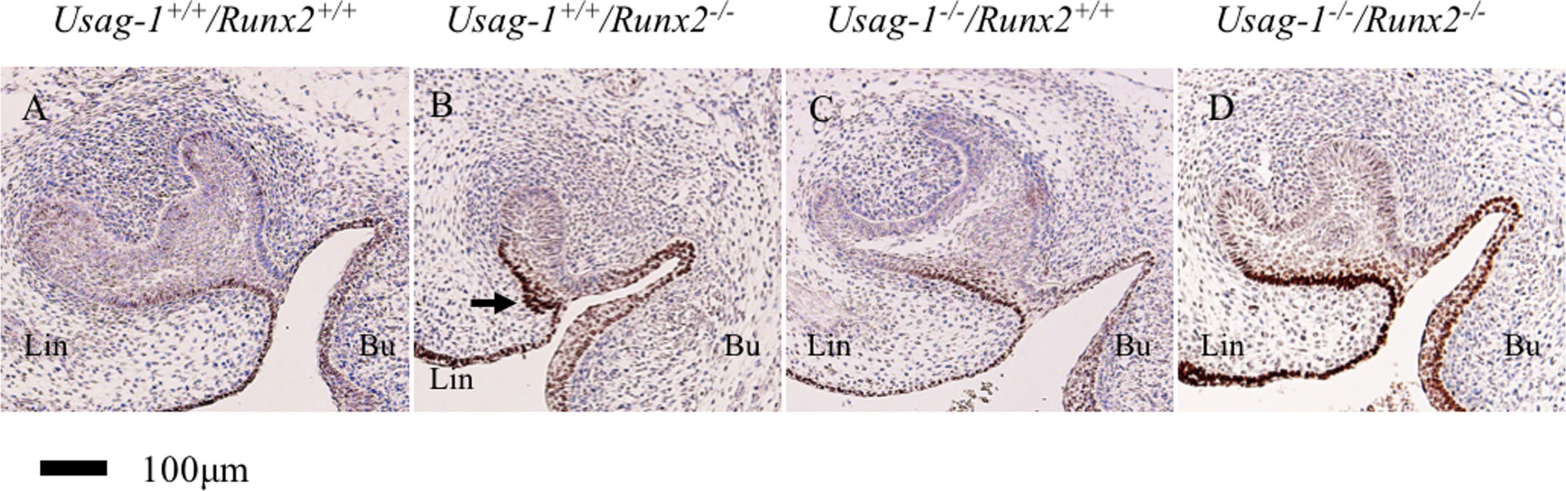
**SOX2 immunostaining in sagittal maxillary molar sections from E15 (A-D) embryos.** At E15, strong SOX2 staining was seen in the lingual bud of the *Usag-1*^*+/+*^/*Runx2*^*-/-*^ mice (B). The arrow indicates the lingual bud that formed in the upper molar of a *Usag-1*^+/+^/*Runx2*^-/-^ mouse (B).

The maxillary incisors displayed hypoplastic phenotypic changes; i.e., supernumerary tooth formation and lingual bud formation, in *Usag-1*- and *Runx2-* deficient mice, respectively. Therefore, in order to investigate the contributions of odontogenic and dental epithelial Sox2-positive cells to these phenotypic changes, we performed immunohistochemical examinations to localize SOX2 in the maxillary incisors of *Usag-1*^+/+^/*Runx2*^+/+^, *Usag-1*^+/+^/*Runx2*^-/-^, *Usag-1*^-/-^/*Runx2*^+/+^ and *Usag-*1^-/-^/*Runx2*^-/-^ mice from E12 through E15 (Fig 6). At E12, faint Sox2 staining was seen within the oral epithelium in every genotype. By E13, Sox2 was localized in the lingual regions of the epithelial thickening and the dental lamina of the maxillary incisors in the *Usag-1*^+/+^/*Runx2*^+/+^, *Usag-1*^-/-^/*Runx2*^+/+^, and *Usag-1*^-/-^/*Runx*2^-/-^ mice (Fig 6E, 6G and 6H). In contrast, in the *Usag-1*^+/+^/*Runx2*^-/-^ mice Sox2 was distributed throughout the entire dental lamina of the incisors (Fig 6F). Over the next two days of development (E14 and E15), Sox2 staining was limited to the lingual side of the incisors in all genotypes (Fig 6I–6P). Interestingly, in the *Usag-1*^+/+^/*Runx2*^-/-^ mice large numbers of Sox2-positive cells were detected in the lingual bud (Fig 6N). Furthermore, Sox2-positive cells were detected in the lingual bud of the maxillary molars (Fig 7B). Sox2 was never detected in the rudimentary incisor region at any stage in any genotype (Fig 6A–6P). These findings suggest that dental epithelial Sox2-positive cells contribute to lingual bud formation, but not to the subsequent development of rudimentary incisors.

## Discussion

In this study, we observed three interesting phenomena in double knockout *Usag-1*^*-/-*^*/Runx2*^*-/-*^ mice: 1) the prevalence of supernumerary teeth was lower in these mice than in *Usag-1*^*-/-*^ mice; 2) tooth development was more advanced in these mice than in *Runx*2^-/-^ mice; and 3) the frequency of maxillary molar lingual buds was lower in these mice than in *Runx2*^*-/-*^ mice (Table 1). These findings suggest that Usag-1 and Runx2 function in an antagonistic manner. Previously, we reported that the inhibition of apoptosis leads to the development of the rudimentary maxillary incisors in *Usag-1* null mice [9]. Furthermore, we suggested that BMP and Wnt signaling work cooperatively to inhibit apoptosis in the odontogenic mesenchymal cells of rudimentary incisors. This indicates that USAG-1 regulates local BMP activity via its functions as a BMP antagonist [19, 42] and a modulator of Wnt signaling [9], resulting in the induction of apoptosis and destruction of the rudimentary incisors [14, 15, 43]. In this study, the prevalence of supernumerary teeth was lower in the double knockout *Usag-1*^*-/-*^*/Runx2*^*-/-*^ mice than in the *Usag-1* null mice (Table 1). These results further suggest that RUNX2 contributes to the progression of rudimentary incisor development via crosstalk between local BMP and Wnt signaling.

**Fig 8:**
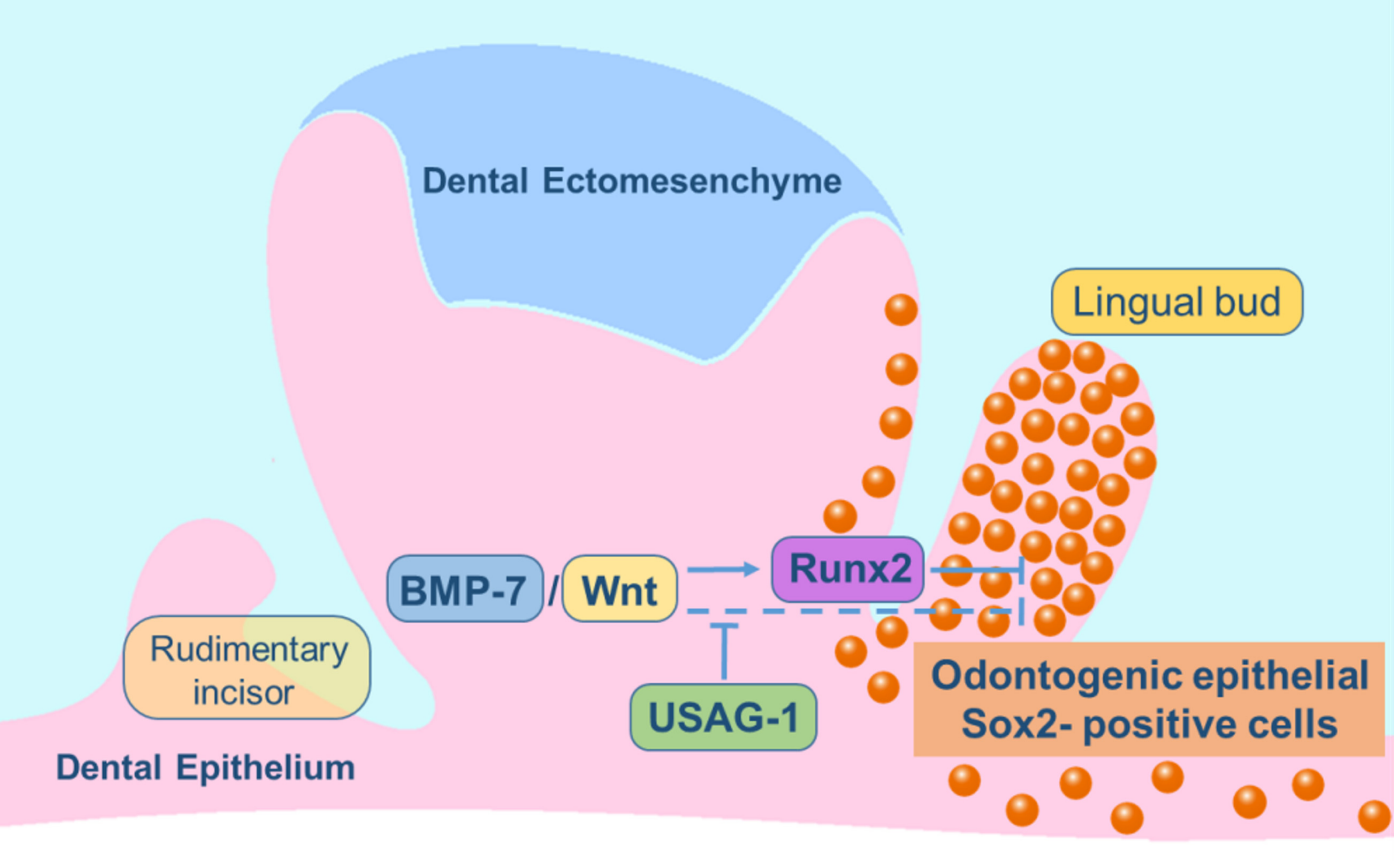
Schematic model of the odontogenic epithelial Sox2-positive cells-associated signaling pathways involved in lingual bud formation in maxillary incisors. Runx2 prevents the differentiation and/or proliferation of odontogenic epithelial Sox2-positive cells. Runx2 is induced by BMP signaling independently of USAG-1, which also inhibits the formation of odontogenic epithelial Sox2-positive cells by acting as a BMP/Wnt antagonist. The orange spheres represent odontogenic epithelial Sox2-positive cells.

Several mechanisms have been proposed to account for supernumerary tooth formation. The most interesting finding of this investigation is that the maxillary incisor lingual bud was almost completely filled with dental epithelial Sox2-positive cells in the *Usag-1*^+/+^/*Runx2*^-/-^ mice (Fig 6N), whereas no odontogenic epithelial Sox2-positive cells contributed to supernumerary tooth formation in the rudimentary maxillary incisors of the *Usag-1*^-/-^/*Runx2*^+/+^ mice (Fig 6O). Previous studies have suggested that the lingual bud formation seen in *Runx2*^-/-^ mice might be representative of initial supernumerary tooth formation, based on the presence of numerous supernumerary teeth in patients with CCD. Furthermore, in the *Runx2*^*-/-*^ mice, deletion of Usag-1 inhibited lingual bud formation during maxillary molar and incisor development. On the contrary, the abrogation of *Runx2* expression prevented supernumerary tooth formation from the rudimentary maxillary incisors in the USAG-1-deficient mice. From an evo-devo viewpoint, the rudimentary maxillary incisors are considered to be a deciduous counterpart of the permanent incisors and are the first generation of teeth [41]. On the other hand, the permanent incisors are the second generation of teeth. Lingual buds from the permanent incisors are considered to be the third generation of teeth because replacement teeth form on the lingual side of the dental arch [36]. This suggests that the mechanism responsible for supernumerary tooth formation differs between *Usag-1-* and *Runx2*-deficient mice. RUNX2 is strongly associated with odontogenic epithelial Sox2-positive cells while USAG-1 is not. It was reported that RUNX2 indirectly contributes to lingual bud formation in odontogenic epithelial cells due to its localization in dental mesenchymal cells at the bell stage of tooth development. RUNX2 is an important transcription factor that regulates incisor development at the late bell stage [27, 30, 44, 45]. Wang et al. (2005) concluded that SHH contributes to successional tooth formation by stimulating dental epithelium budding and cell proliferation and that RUNX2 inhibits SHH expression [29]. However, there is no evidence to support the direct induction of lingual budding by SHH. Furthermore, the localization of RUNX2 in dental mesenchymal cells was examined using *in situ* hybridization [27, 45]. Due to technical limitations regarding the sensitivity of this technique, it is unclear whether RUNX2 is expressed in odontogenic epithelial cells at the bell stage of tooth development. *Runx* genes have been reported to be involved in the maintenance of epithelial stem cells in mouse incisors [46]. It has clearly been shown that the three Runx2 isoforms have different functions and temporospatial expression patterns during osteogenesis [33]. In order to confirm the expression patterns of Runx2 in odontogenic epithelial cells, we investigated the expression of the three Runx2 isoforms in mHAT9d cells [47], which are derived from the dental epithelial tissue of the mouse incisor apical bud, using the semi-quantitative reverse transcription polymerase chain reaction. We have shown that the three Runx2 isoforms, types I, II, and III, all are expressed, as are Runx1 and Runx3 S1 Fig. As for odontogenesis, it has been suggested that type II and III Runx2 have different functions from type I in this process [33]. Furthermore, during amelogenesis, which involves the differentiation of the inner enamel epithelial cells to enamel-producing ameloblasts, the various isoforms of Runx2 were expressed observed at different developmental stages of ameloblast cytodifferentiation [32, 44]. Only type I Runx2 was expressed by secretory ameloblasts, suggesting that type II or III might directly regulate the differentiation and/or proliferation of odontogenic epithelial stem cells. Based on our findings, we propose a genetic pathway that might contribute to odontogenic epithelial Sox2-positive cell formation. Since Runx2 is a downstream target gene of BMP signaling [44], it might prevent the differentiation or proliferation of odontogenic epithelial Sox2-positive cells directly or indirectly. Therefore, we hypothesized that Runx2 expression is induced by BMP signaling independently of Usag-1 (Fig 8).

*Runx2*^-/-^ mice exhibit stunted tooth formation. Recently, a patient with a unique Arg131Cys missense *RUNX2* mutation was shown to have a novel dental phenotype; i.e., no supernumerary teeth but one congenitally missing tooth [48]. In this study, we demonstrated that the deletion of *Usag-1* rescued the hypoplastic and poorly differentiated molar and incisor phenotypes seen in *Runx2*^-/-^ mice. The rescue of tooth formation in genetically defined mouse models clearly demonstrates the feasibility of inducing *de novo* tooth formation via the *in situ* repression of a single targeted gene. Our investigations and related studies clearly validate the hypothesis that the *de novo* repression of target genes, such as *Usag-1*, could be used to stimulate arrested tooth germs in order to induce new tooth formation in mammals. Molecular targeted therapy is a type of treatment in which drugs or other substances are used to specifically attack certain cell types by interfering with critical target molecules. Indeed, in animal models of ectodysplasin A (EDA) deficiency, which is associated with the human disorder hypohidrotic ectodermal dysplasia (HED) (which involves hypodontia), the administration of a soluble EDA receptor agonist has been shown to correct many phenotypic abnormalities, including abnormalities of the dentition in mice (primary) and dogs (secondary or permanent) [49–52]. In fact, lifelong phenotypic correction was achieved after a rather short course of treatment [49, 52]. Molecular targeted therapy could be used to generate teeth in patients with congenital tooth agenesis by stimulating arrested tooth germs.

## Conclusion

We suggest that RUNX2 and USAG-1 function in an antagonistic manner during tooth formation. The lingual bud was filled with odontogenic epithelial Sox2-positive cells in *Usag-1*^+/+^/*Runx2*^-/-^ mice, whereas almost no odontogenic epithelial Sox2-positive cells contributed to supernumerary tooth formation in the rudimentary maxillary incisors of *Usag-1*^-/-^/*Runx2*^+/+^ mice. Our findings indicate that RUNX2 directly or indirectly prevents the differentiation or proliferation of odontogenic epithelial Sox2-positive cells. We hypothesize that RUNX2 inhibits the BMP and/or Wnt signaling pathways regulated by USAG-1, whereas Runx2 expression is induced by BMP signaling independently of USAG-1.

## Supporting Information

S1 Fig Semi-quantitative RT-RCR analysis of Runx family and Runx2 expression in mHAT9d. RNA was purified from the mHAT9d cells. RT products were thirty-fold and ninety-fold serially diluted and subjected to PCR. Reduced glyceraldehyde-phosphate dehydrogenase (GAPDH) was used as an internal control.
(TIF)

S1 Table RT-PCR Oligonucleotide Primers Used to Determine mRNA Expression Levels. (DOC)

## References

[1] Zhou P, Byrne C, Jacobs J, Fuchs E. Lymphoid enhancer factor 1 directs hair follicle patterning and epithelial cell fate. Genes Dev. 1995;9: 700–713. 7537238 10.1101/gad.9.6.700

[2] Kaufman MH, Chang HH Shaw JP. Craniofacial abnormalities in homozygous Small eye (Sey/Sey) embryos and newborn mice. J Anat. 1995; 186: 607–617. 7559133 PMC1167018

[3] Mustonen T, Pispa J, Mikkola ML, Pummila M, Kangas AT, Pakkasjärvi L, et al Stimulation of ectodermal organ development by Ectodysplasin-A1. Dev Biol. 2003;259: 123–136. 12812793 10.1016/s0012-1606(03)00157-x

[4] Zhang Q, Murcia NS, Chittenden LR, Richards WG, Michaud EJ, Woychik RP, et al Loss of the Tg737 protein results in skeletal patterning defects. Dev Dyn. 2003;227: 78–90. 12701101 10.1002/dvdy.10289

[5] Pispa J, Mustonen T, Mikkola ML, Kangas AT, Koppinen P, Lukinmaa PL, et al Tooth patterning and enamel formation can be manipulated by misexpression of TNF receptor Edar. Dev dyn. 2004;231: 432–440. 15366021 10.1002/dvdy.20138

[6] Tucker AS, Headon DJ, Courtney JM, Overbeek P, Sharpe PT. The activation level of the TNF family receptor, Edar, determines cusp number and tooth number during tooth development. Dev Biol. 2004;268: 185–194. 15031115 10.1016/j.ydbio.2003.12.019

[7] Klein OD, Minowada G, Peterkova R, Kangas A, Yu BD, Lesot H, et al Sprouty genes control diastema tooth development via bidirectional antagonism of epithelial-mesenchymal FGF signaling. Dev Cell. 2006;11: 181–190. 16890158 10.1016/j.devcel.2006.05.014 PMC2847684

[8] Järvinen E, Salazar-Ciudad I, Birchmeier W, Taketo MM, Jernvall J, Thesleff I. Continuous tooth generation in mouse is induced by activated epithelial Wnt/beta-catenin signaling. Proc Nati Acad Sci (USA). 2006;103: 18627–18632. 10.1073/pnas.0607289103 PMC1693713 17121988

[9] Murashima-Suginami A, Takahashi K, Kawabata T, Sakata T, Tsukamoto H, Sugai M, et al Rudiment incisors survive and erupt as supernumerary teeth as a result of USAG-1 abrogation. Biochem Biophys Res Commun. 2007;359: 549–555. 17555714 10.1016/j.bbrc.2007.05.148

[10] Nakamura T, de Vega S, Fukumoto S, Jimenez L, Unda F, Yamada Y. Transcription factor epiprofin is essential for tooth morphogenesis by regulating epithelial cell fate and tooth number. J Biol Chem. 2008;283: 4825–4833. 18156176 10.1074/jbc.M708388200

[11] Ohazama A, Johnson EB, Ota MS, Choi HY, Porntaveetus T, Oommen S, et al (2008). Lrp4 modulates extracellular integration of cell signaling pathways in development. PLoS One 3: e4092 10.1371/journal.pone.0004092 19116665 PMC2605561

[12] Zhang Z, Lan Y, Chai Y, Jiang R. Antagonistic actions of Msx1 and Osr2 pattern mammalian teeth into a single row. Science. 2009;323: 1232–1234. 10.1126/science.1167418 19251632 PMC2650836

[13] Huang B, Takahashi K, Sakata-Goto T, Kiso H, Togo Y, Saito K, et al Phenotypes of CCAAT/enhancer-binding protein beta deficiency: hyperdontia and elongated coronoid process. Oral Dis. 2013;19: 144–150. 10.1111/j.1601-0825.2012.01963.x 22849712

[14] Takahashi K, Kiso H, Saito K, Togo Y, Tsukamoto H, Huang B, et al Feasibility of gene therapy for tooth regeneration by stimulation of a third dentition In: Francisco M, editors. Gene therapy: tools and potential applications. Croatia: InTech, Rijeka; 2013 pp. 727–744.

[15] Murashima-Suginami A, Takahashi K, Sakata T, Tsukamoto H, Sugai M, Yanagita M, et al Enhanced BMP signaling results in supernumerary tooth formation in USAG-1 deficient mouse. Biochem Biophys Res Commun. 2008;369: 1012–1016. 10.1016/j.bbrc.2008.02.135 18329379

[16] Wang XP, O'Connell DJ, Lund JJ, Saadi I, Kuraguchi M, Turbe-Doan A, et al Apc inhibition of Wnt signaling regulates supernumerary tooth formation during embryogenesis and throughout adulthood. Development. 2009;136: 1939–1949. 10.1242/dev.033803 19429790 PMC2680115

[17] Yamamoto H, Cho SW, Song SJ, Hwang HJ, Lee MJ, Kim JY, et al Characteristic tissue interaction of the diastema region in mice. Arch Oral Biol. 2005;50: 189–198. 15812993 10.1016/j.archoralbio.2004.11.010

[18] Lagronova-Churava S, Spoutil F, Vojtechova S, Lesot H, Peterka M, Klein OD, et al The dynamics of supernumerary tooth development are differentially regulated by Sprouty genes. J Exp Zool B Mol Dev evol. 2013;320: 307–320. 10.1002/jez.b.22502 23606267

[19] Yanagita M, Oka M, Watabe T, Iguchi H, Niida A, Takahashi S, et al USAG-1: a bone morphogenetic protein antagonist abundantly expressed in the kidney. Biochem Biophys Res Commun. 2004;316: 490–500. 15020244 10.1016/j.bbrc.2004.02.075

[20] Yanagita M, Okuda T, Endo S, Tanaka M, Takahashi K, Sugiyama F, et al Uterine sensitization-associated gene-1 (USAG-1), a novel BMP antagonist expressed in the kidney, accelerates tubular injury. J Clin Invsest. 2006;116: 70–79. 10.1172/JCI25445 PMC1307562 16341262

[21] Yanagita M. Modulator of bone morphogenetic protein activity in the progression of kidney diseases. Kidney Int. 2006;70: 989–993. 16871237 10.1038/sj.ki.5001731

[22] Kiso H, Takahashi K, Saito K, Togo Y, Tsukamoto H, Huang B, et al (2014). Interactions between BMP-7 and USAG-1 (uterine sensitization-associated gene-1) regulate supernumerary organ formations. PLoS One 9: e96938 10.1371/journal.pone.0096938 24816837 PMC4016158

[23] Fleischmannova J, Matalova E, Tucker AS, Sharpe PT. Mouse models of tooth abnormalities. Eur J Oral Sci. 2008;116(1): 1–10. 10.1111/j.1600-0722.2007.00504.x 18186725

[24] Peters H, Neubüser A, Kratochwil K, Balling R. Pax9-deficient mice lack pharyngeal pouch derivatives and teeth and exhibit craniofacial and limb abnormalities. Genes Dev. 1998;12: 2735–47. 9732271 10.1101/gad.12.17.2735 PMC317134

[25] Satokata I, Maas R. Msx1 deficient mice exhibit cleft palate and abnormalities of craniofacial and tooth development. Nat Genet. 1994;6: 348–56. 7914451 10.1038/ng0494-348

[26] van Genderen C, Okamura RM, Fariñas I, Quo RG, Parslow TG, Bruhn L, et al Development of several organs that require inductive epithelia-mesenchymal interactions in impaired in LEF-1-deficient mice. Genes Dev. 1994;8; 2691–2703. 7958926 10.1101/gad.8.22.2691

[27] D'Souza RN, Aberg T, Gaikwad J, Cavender A, Owen M, Karsenty G, et al Cbfa1 is required for epithelial-mesenchymal interactions regulating tooth development in mice. Development. 1999;126: 2911–2920. 10357935 10.1242/dev.126.13.2911

[28] Mundlos S, Otto F, Mundlos C, Mulliken JB, Aylsworth AS, Albright S, et al Mutations involving the transcription factor CBFA1 cause cleidocranial dysplasia. Cell. 1997;30:773–779. 10.1016/s0092-8674(00)80260-3 9182765

[29] Wang XP, Aberg T, James MJ, Levanon D, Groner Y, Thesleff I. Runx2(Cbfa1) inhibits Shh signaling in the lower but not upper molars of mouse embryos and prevents the budding of putative successional teeth. J Dent Res. 2005;84: 138–143. 15668330 10.1177/154405910508400206

[30] Aberg T, Cavender A, Gaikwad JS, Bronckers AL, Wang X, Waltimo-Sirén J, et al Phenotypic changes in dentition of Runx2 Homozygote-null mutant mice. J Histochem Cytochem. 2004;52: 131–139. 14688224 10.1177/002215540405200113

[31] Kassai Y, Munne P, Hotta Y, Penttilä E, Kavanagh K, Ohbayashi N, et al Regulation of mammalian tooth cusp patterning by ectodin. Science. 2005;23:2067–2070. 10.1126/science.1116848 16179481

[32] Chen S, Gu TT, Sreenath T, Kulkarni AB, Karsenty G, MacDougall M. Spatial expression of Cbfa1/Runx2 isoforms in teeth and characterization of binding sites in the DSPP gene. Connect Tissue Res. 2002;43: 338–344. 12489178 10.1080/03008200290000691

[33] Kobayashi I, Kiyoshima T, Wada H, Matsuo K, Nonaka K, Honda JY, et al Type II/III Runx2/Cbfa1 is required for tooth germ development. Bone. 2006;38: 836–844. 16377268 10.1016/j.bone.2005.10.026

[34] Jiang H, Sodek J, Karsenty G, Thomas H, Ranly D, Chen J. Expression of core binding factor Osf2/Cbfa-1 and bone sialoprotein in tooth development. Mech Dev. 1999;81: 169–173. 10330494 10.1016/s0925-4773(98)00232-9

[35] Zhang L, Yuan G, Liu H, Lin H, Wan C, Chen Z. Expression pattern of Sox2 during mouse tooth development. Gene Expr Patterns. 2012;12: 273–281. 10.1016/j.gep.2012.07.001 22835638

[36] Juuri E, Saito K, Ahtiainen L, Seidel K, Tummers M, Hochedlinger K, et al Sox2+ stem cells contribute to all epithelial lineages of the tooth via Sfrp5+ progenitors. Dev Cell. 2012;23: 317–328. 10.1016/j.devcel.2012.05.012 22819339 PMC3690347

[37] Juuri E, Jussila M, Seidel K, Holmes S, Wu P, Richman J, et al Sox2 marks epithelial competence to generate teeth in mammals and reptiles. Development. 2013;140: 1424–1432. 10.1242/dev.089599 23462476 PMC3596986

[38] Wang XP, Suomalainen M, Felszeghy S, Zelarayan LC, Alonso MT, Plikus MV, et al (2007). An integrated gene regulatory network controls stem cell proliferation in teeth. PLoS Biol 25: e159. 10.1371/journal.pbio.0050159 PMC1885832 17564495

[39] Komori T, Yagi H, Nomura S, Yamaguchi A, Sasaki K, Deguchi K, et al Targeted disruption of Cbfa1 results in a complete lack of bone formation owing to maturational arrest of osteoblasts. Cell. 1997;89: 755–764. 9182763 10.1016/s0092-8674(00)80258-5

[40] Ducy P, Zhang R, Geoffroy V, Ridall AL, Karsenty G. Osf2/Cbfa1: a transcriptional activator of osteoblast differentiation. Cell. 1997;89: 747–754. 9182762 10.1016/s0092-8674(00)80257-3

[41] Fitzgerald LR. Deciduous incisor teeth of the mouse (Mus musculus). Arch Oral Biol. 1973;18: 381–389. 4515966 10.1016/0003-9969(73)90162-3

[42] Laurikkala J, Kassai Y, Pakkasjärvi L, Thesleff I, Itoh N. Identification of a secreted BMP antagonist ectodin, integrating BMP, FGF, and SHH signals from the tooth enamel knot. Dev Biol. 2003;64: 91–105. 10.1016/j.ydbio.2003.08.011 14623234

[43] Itasaki N, Jones CM, Mercurio S, Rowe A, Domingos PM, Smith JC, et al Wise, a context-dependent activator and inhibitor of Wnt signalling. Development. 2003;130: 4295–4305. 12900447 10.1242/dev.00674

[44] Camilleri S, McDonald F. Runx2 and dental development. Eur J Oral Sci. 2006;114: 361–373. 17026500 10.1111/j.1600-0722.2006.00399.x

[45] Chen S, Gluhak-Heinrich J, Wang YH, Wu YM, Chuang HH, Chen L, et al Runx2, osx, and dspp in tooth development. J Dent Res. 2009;88: 904–909. 10.1177/0022034509342873 19783797 PMC3045537

[46] Kurosaka H, Islam MN, Kuremoto K, Hayano S, Nakamura M, Kawanabe N, et al Core binding factor beta functions in the maintenance of stem cells and orchestrates continuous proliferation and differentiation in mouse incisors. Stem Cells. 2011;29: 1792–1803. 10.1002/stem.722 21898689

[47] Otsu K, Kishigami R, Fujiwara N, Ishizeki K, Harada H. Functional role of Rho-kinase in ameloblast differentiation. J Cell Physiol. 2011;226: 2527–2534. 10.1002/jcp.22597 21792909

[48] Callea M, Fattori F, Yavuz I, Bertini E. (2012). A new phenotypic variant in cleidocranial dysplasia (CCD) associated with mutation c.391C>T of the RUNX2 gene. BMJ Case Rep 5. 10.1136/bcr-12-2011-5422 PMC4542990 23220435

[49] Gaide O, Schneider P. Permanent correction of an inherited ectodermal dysplasia with recombinant EDA. Nat Med. 2003; 9: 614–618. 12692542 10.1038/nm861

[50] Casal ML, Lewis JR, Mauldin EA, Tardivel A, Ingold K, Favre M, et al Significant correction of disease after postnatal administration of recombinant ectodysplasin A in canine X-linked ectodermal dysplasia. Am J Hum Genet, 2007;81: 1050–1056. 17924345 10.1086/521988 PMC2265652

[51] Mauldin EA, Gaide O, Schneider P, Casal ML. Neonatal treatment with recombinant ectodysplasin prevents respiratory disease in dogs with X-linked ectodermal dysplasia. Am J Med Genet A. 2009;149A: 2045–2049. 10.1002/ajmg.a.32916 19533784 PMC2754310

[52] Kowalczyk C, Dunkel N, Willen L, Casal ML, Mauldin EA, Gaide O, et al Molecular and therapeutic characterization of anti-ectodysplasin A receptor (EDAR) agonist monoclonal antibodies. J Biol Chem. 2001;286: 30769–30779. 10.1074/jbc.M111.267997 PMC3162438 21730053

