# Supplementary figures and images for: Antagonistic Functions of USAG-1 and RUNX2 during Tooth Development
Source: PLoS One. 2016 Aug 12;11(8):e0161067. doi: 10.1371/journal.pone.0161067 (PMC4982599; doi:10.1371/journal.pone.0161067)

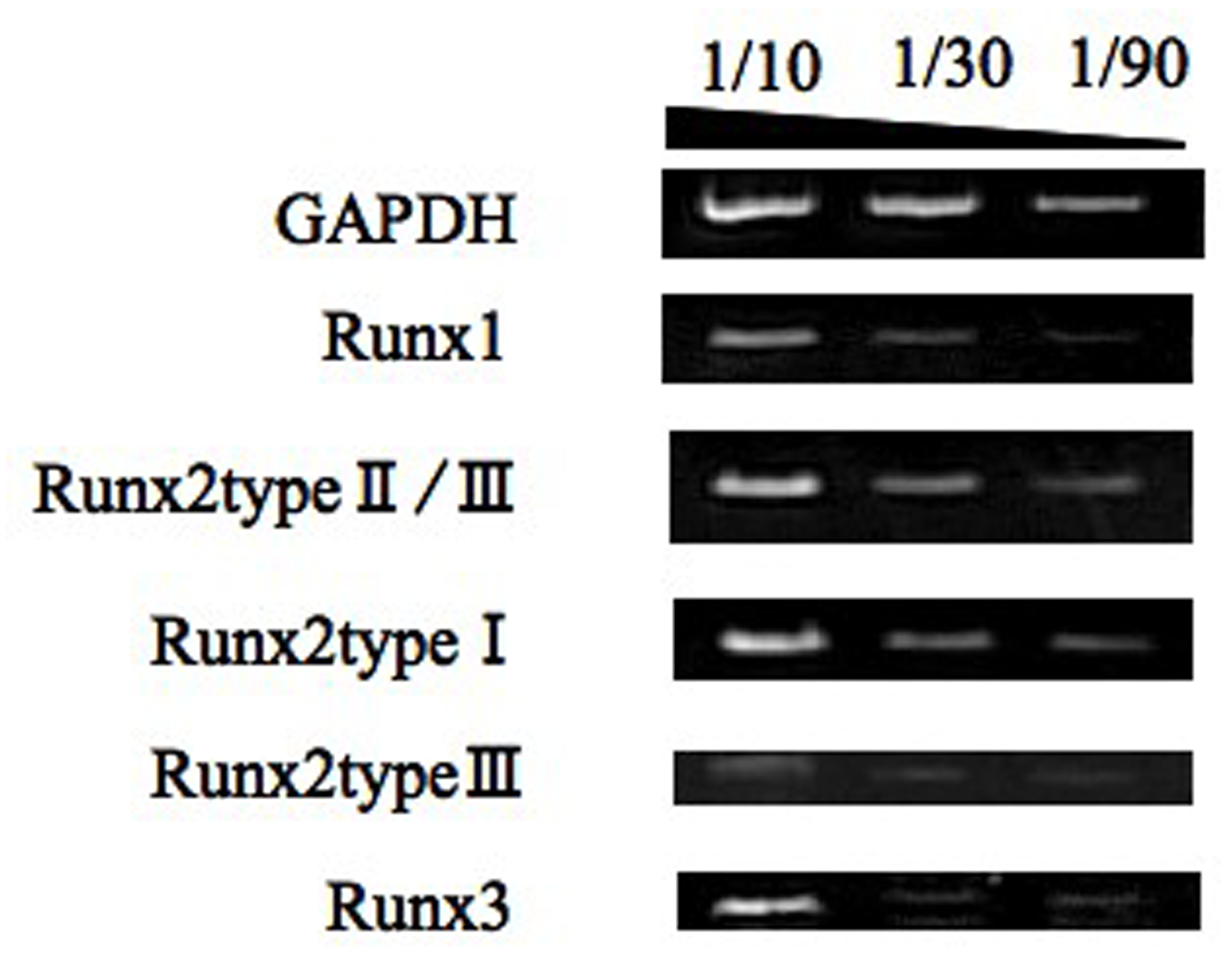

Supplement: S1 Fig — RNA was purified from the mHAT9d cells. RT products were thirty-fold and ninety-fold serially diluted and subjected to PCR. Reduced glyceraldehyde-phosphate dehydrogenase (GAPDH) was used as an internal control. (TIF) [file pone.0161067.s001.tif]
