# Supplementary material for: Antagonistic Functions of USAG-1 and RUNX2 during Tooth Development
Source: PLoS One. 2016 Aug 12;11(8):e0161067. doi: 10.1371/journal.pone.0161067 (PMC4982599; doi:10.1371/journal.pone.0161067)

Supplemental material

**Material and methods**

Cell culture

mHAT9d cells are a dental epithelial cell line derived from the apical bud of a mouse incisor. Culture medium consisted of Dulbecco’s modified Eagle’s medium/F12 (GIBCO) containing B-27 supplement (GIBCO), bFGF (25ng/ml; R&D Systems, Minneapolis, MN), and EGF (100g/ml;R&DSystems) and penicillin streptmysin(1%). These cells were cultured on the Primaria(BD Falcon).

Semi-quantitive reverese transcription polymerase chain reaction(RT-PCR)

Total RNA in the 70% confluent mHAT9d cell line was extracted using a TRIZOL reagent(Invitrogen), 0.2ml of chloroform and 0.2ml of phenol choloroform per 1 ml of TRIZOL. The RNA was precipitated from the aqueous phase by mixing with isopropyl alcohol and 16.7 mg/ml glycogen. Reverse transcription of total RNA(3μg) was synthesized using a SuperScript ®ⅢFirst-Strand Synthesis System(Invitrogen).

150ng cDNA of cDNA was serially diluted and PCR amplification was performed using Ex Taq (Takara Bio GAPDH) and KOD FX(the others, TOYOBO) and specific oligonucleotide primers for target sequences(Table 1). All PCR products were examined employing an electrophoretical technique that used 2% agarose gel and ethidium bromide staining. These bands were quantitated with a Bio-image analyzer (FAS-Ⅳ, NIPPON Genetics, Tokyo, Japan). Experiments were carried out in triplicate.

**Result**

Runx2 consists of three different isoforms, which involves type I, II and III. We found all three isoforms of Runx2 were expressed in mHAT9d cells in addition to Runx1 and Runx3.

**Figure legends**

S1 Fig. Semi-quantitative RT-RCR analysis of Runx family and Runx2 expression in mHAT9d. RNA was purified from the mHAT9d cells. RT products were thirty-fold and ninety-fold serially diluted and subjected to PCR. Reduced glyceraldehyde-phosphate dehydrogenase (GAPDH) was used as an internal control.

S1 Table. RT-PCR Oligonucleotide Primers Used to Determine mRNA Expression Levels


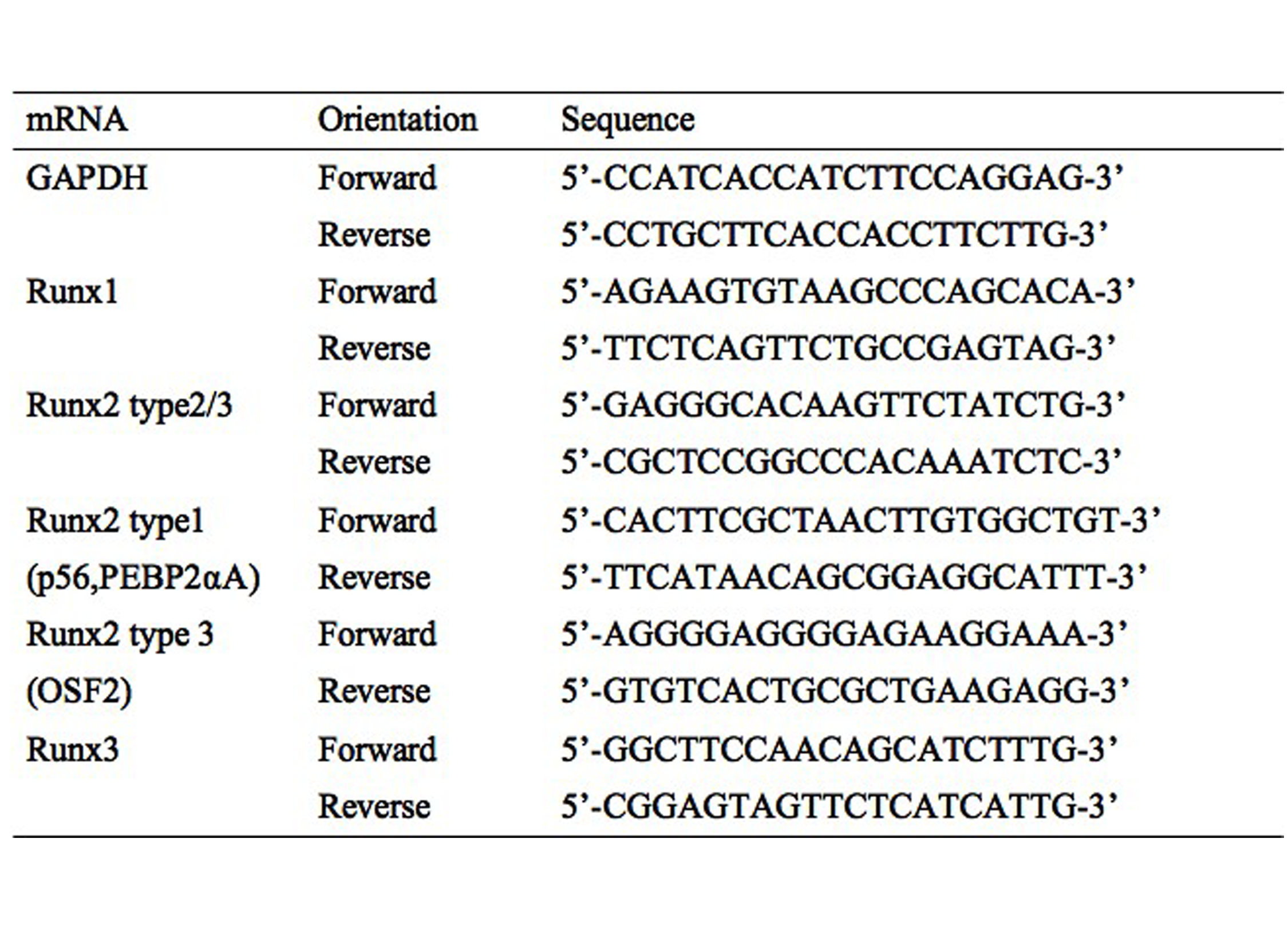

Supplement: S1 Table — (DOC) [file pone.0161067.s002.doc]
